# Frailty and sarcopenia as independent predictors of early functional recovery in older adults with osteoporotic vertebral compression fractures: a retrospective cohort study

**DOI:** 10.3389/fnut.2026.1841245

**Published:** 2026-06-09

**Authors:** Jinxian Liu, Hui Li, Yanyan Su, Xiaoyu Xue, Jianguo Zhang, Lizhu Zhang, Juan Xu

**Affiliations:** 1 Department of Orthopedics, Shanxi Bethune Hospital, Shanxi Academy of Medical Sciences, Third Hospital of Shanxi Medical University, Tongji Shanxi Hospital, Taiyuan, China; 2 Department of Geriatric Medicine, Shanxi Bethune Hospital, Shanxi Academy of Medical Sciences, Third Hospital of Shanxi Medical University, Tongji Shanxi Hospital, Taiyuan, China; 3 Department of Radiotherapy, Shanxi Bethune Hospital, Shanxi Academy of Medical Sciences, Third Hospital of Shanxi Medical University, Tongji Shanxi Hospital, Taiyuan, China; 4 Department of Hepatobiliary Surgery, Shanxi Bethune Hospital, Shanxi Academy of Medical Sciences, Third Hospital of Shanxi Medical University, Tongji Shanxi Hospital, Taiyuan, China

**Keywords:** frailty, functional recovery, muscle health, nutritional status, osteoporotic vertebral fracture, sarcopenia

## Abstract

**Background:**

The high burden of functional and chronic disability in older adults due to osteoporotic vertebral compression fractures (OVCFs) reflects both fracture characteristics and patient-related factors influencing recovery. The underlying conditions that define frailty and sarcopenia, or the inability of osteoporotic patients to maintain muscle mass, may have an important role in functional recovery after OVCF.

**Methods:**

In this retrospective cohort study, 120 patients aged ≥60 years who were diagnosed with radiologically confirmed OVCFs and underwent treatment in a tertiary care center from January 2020 to December 2023 were included. Patients were stratified based on sarcopenia and frailty. The primary outcome of interest was functional recovery, as measured by the Barthel Index at discharge; secondary outcomes included length of hospital stay, complications, VAS pain score, and hospital-based complications. Multivariable linear regression of functional recovery on the independent variables was then calculated. The primary endpoint reflects early in-hospital functional recovery and does not capture long-term outcomes.

**Results:**

Compared to non-frail patients, frail individuals had lower Barthel Index scores (65.3 ± 12.4 vs. 82.5 ± 10.2, *p* < 0.001), higher pain (5.1 ± 1.5 vs. 3.8 ± 1.2, *p* = 0.02), longer length of stay (12.4 ± 4.1 vs. 8.5 ± 3.2 days, *p* = 0.01), and higher complication rates (27.7% vs. 10.9%, *p* = 0.03). Similar patterns were observed among patients with sarcopenia. Multivariable regression models showed that frailty (β = −12.45, *p* < 0.001) and sarcopenia (β = −8.32, *p* = 0.004) were independently associated with reduced early functional recovery.

**Conclusion:**

Frailty and sarcopenia were independently associated with reduced early functional recovery in older adults with osteoporotic vertebral compression fractures. These findings highlight the potential importance of incorporating geriatric and muscle health assessments into clinical evaluation; however, further prospective studies are required to confirm these associations.

## 1 Introduction

Compression fractures of osteoporotic vertebrae (OVCFs) are among the most common fragility fractures in aged people. They represent a major source of morbidity, functional decline, and lower quality of life worldwide (1–6). Osteoporotic vertebral fractures are a type of fragility fracture resulting from low-energy trauma due to low bone mineral density. Increased OVCF prevalence in an aging population is a significant burden for health care systems and LTC resources, including higher hospitalization rates, long-term disability, and increased costs (1–8).

OVCFs are associated with both acute and chronic pain; loss of mobility; spinal deformities; and an increased risk for future fractures leading to ongoing disability and increased dependence (2–6, 9–12). There has been little improvement in OVCF outcomes over time despite advances in imaging technology and treatment strategies, and outcomes are variable across older patients. Historically, clinical evaluation of OVCFs has primarily focused on the structural features of the fracture and radiographic evidence of severity. Ongoing evidence indicates that recovery from an OVCF is primarily determined by patient-specific factors, such as physiologic reserve and musculoskeletal health. Thus, OVCF cases should be viewed as part of a broader geriatric picture rather than merely as skeletal conditions. Frailty is a syndrome characterized by multiple factors that reduce the body's capacity to cope with stressors, resulting from diminished metabolic or physiological reserve. Frailty is one of the most significant risk factors for adverse outcomes in older adults (13–15). Older, frail individuals typically have a higher likelihood of their functional ability declining, experiencing longer lengths of stay in hospitals, and high rates of complications from acute illness/injury (13–15). Within the population of individuals with osteoporotic fractures, frail individuals have higher rates of fracture, less successful recovery, and greater mortality (13–15).

Sarcopenia is the age-associated decline in both lean muscle mass and strength, making it another major age-related disorder. Sarcopenia has been shown to significantly correlate with greater impairment of functional capacity and poor clinical outcomes (16–18). The development of postural instability and functional impairment as a result of vertebral fracture may be exacerbated by underlying sarcopenia, as sarcopenia limits rehabilitation and recovery (16–18). In addition, sarcopenia is increasingly being recognized as a disorder influenced by the interaction of nutritional status and metabolic factors, including inadequate dietary protein intake, altered energy balance, and systemic inflammation, all of which may restrict the ability of elderly individuals to have functional recovery (16–18).

Frailty and sarcopenia are frequently coexisting comorbid conditions that can exacerbate each other's effects. Frailty indicates general physiological vulnerability, while sarcopenia reflects reduced muscle structure and function. The compounded effects of these two disorders significantly increase the likelihood of poor functional recovery after an osteoporotic fracture (13–18).

While both frailty and sarcopenia have been thoroughly researched among individuals with hip fractures, it is less certain what effect they have on the early functional recovery and inpatient outcomes after osteoporotic vertebral compression fractures (OVCFs). Unlike hip fractures, OVCFs can present with a heterogeneous array of clinical presentations due to spinal deformity and associated pain and mobility limitations, thereby requiring a more specific evaluation tailored to each patient's condition.

Despite the increasing focus that has been given to frailty and sarcopenia, there remains a lack of clarity surrounding the combined and individual contributions of these disorders to the functional recovery of elderly persons following OVCFs. Therefore, the purpose of this study is to examine the relationships between frailty and sarcopenia, and between each and functional recovery, in OVCF patients. Specifically, we sought to identify the relationship between frailty and sarcopenia and functional independence, length of stay, complications, and pain outcomes in OVCF patients, and to assess whether frailty and sarcopenia have independent effects using multivariable regression. We hope that the results of this study may provide insight into improved clinical assessments and risk stratification in elderly OVCF patients.

## 2 Materials and methods

### 2.1 Study design and setting

The Department of Orthopedics at Shanxi Bethune Hospital, Shanxi Academy of Medical Sciences, and the Third Hospital of Shanxi Medical University conducted this retrospective cohort study. We screened consecutive patients coming in for treatment of osteoporotic vertebral compression fractures (OVCFs) between January 2020 and December 2023 to determine eligibility for inclusion in our study. This study was conducted and reported in accordance with the Strengthening the Reporting of Observational Studies in Epidemiology (STROBE) guidelines to enhance transparency and reproducibility of the methods used.

### 2.2 Study population

Eligibility criteria included patients aged 60 years or older with radiologically confirmed osteoporotic vertebral compression fractures who received standardized inpatient management, including analgesia, mobilization support, and, where indicated, vertebral augmentation procedures, at a single institution during the study period.

Exclusion criteria included having a fracture due to high-energy trauma, a pathological fracture due to malignancy/infection, severe pre-existing neurological deficits, a history of prior spine surgery, or incomplete clinical data prohibiting evaluation of outcomes.

The final analysis comprised 120 patients after applying the aforementioned inclusion and exclusion criteria.

### 2.3 Data collection

Using an established data collection form, the clinical data were extracted from a retrospective cohort of hospital electronic medical record systems. Data were also collected on demographic variables (age, sex, BMI) and population co-morbidities (hypertension, diabetes mellitus, cardiovascular disease, and other chronic conditions).

Comorbidities were included as a single cumulative variable representing the total number of chronic conditions per patient, including hypertension, diabetes mellitus, and cardiovascular disease.

Fracture-related variables included fracture types, the number of affected vertebral levels, and imaging characteristics. Treatment-related variables were divided into whether conservative or augmentation treatment was used, whether analgesia was used, and, where applicable, perioperative factors.

The treatment modality (conservative vs. vertebral augmentation) was recorded and examined in supplementary analyses; however, inclusion in the multivariable model was limited by sample size constraints and the risk of overfitting. Importantly, exploratory inclusion of this variable did not materially alter the direction or statistical significance of the observed associations.

Rehabilitation-related variables included early mobilization and physiotherapy interventions performed during hospitalization, delivered by trained rehabilitation personnel, including physiotherapists, as part of standard inpatient care.

Laboratory indicators, including albumin levels, were collected as markers of nutritional status. In addition, standardized nursing assessments were performed to evaluate functional status, mobility, and nutritional risk during hospitalization. Although albumin was recorded as a marker of nutritional status, incomplete data availability precluded its inclusion in the baseline comparative analysis. Physiotherapy interventions were delivered by trained rehabilitation personnel, including physiotherapists, as part of standard inpatient care.

### 2.4 Assessment of frailty and sarcopenia

Frailty was determined from routinely recorded clinical indicators aligned with the Clinical Frailty Scale (CFS) framework, including functional dependence, mobility limitation, and comorbidity burden. Where direct CFS scoring was unavailable, patients were classified based on documented multidisciplinary clinical assessments reflecting frailty characteristics.

For sarcopenia, definitions were developed in accordance with the principles of the European Working Group on Sarcopenia in Older People (EWGSOP2). Measures included muscle strength, physical performance, and/or documented muscle loss. In cases where direct quantitative measurements (e.g., grip strength or imaging) were unavailable, surrogate indicators were used, including documented mobility limitation, dependence in activities of daily living, clinically assessed muscle weakness, and nutritional risk, as recorded in standardized nursing and rehabilitation assessments. Importantly, dependence in activities of daily living, used as a surrogate indicator of sarcopenia, was derived from routine clinical documentation rather than the Barthel Index, which served as the primary outcome measure, thereby minimizing potential circularity in outcome assessment.

Patients were categorized according to frailty status (frail vs. non-frail) and sarcopenia status (sarcopenic vs. non-sarcopenic) to enable evaluation of their independent effects on clinical outcomes. Among patients classified as having sarcopenia, both EWGSOP2-aligned objective criteria and clinically documented surrogate indicators were utilized. Due to the dataset's retrospective nature and variability in clinical documentation, precise subgroup counts could not be reliably extracted. However, inclusion of both approaches was intended to reflect real-world clinical practice and enhance external validity. This pragmatic classification approach was adopted to reflect real-world clinical practice, where standardized sarcopenia assessments are not consistently performed in routine inpatient settings. Future prospective studies using standardized and uniformly applied sarcopenia assessment criteria are warranted to further validate these findings. Although precise subgroup counts could not be extracted, most sarcopenia classifications were based on clinically documented functional and mobility impairments, consistent with real-world inpatient assessment practices.

### 2.5 Outcome measures

Functional recovery at discharge was the primary endpoint of this study, as measured by the Barthel Index, a validated tool for measuring activities of daily living and functional independence. The secondary endpoints included pain level measured by the Visual Analog Scale (VAS), length of hospitalization, in-hospital complications (including, but not limited to, infection, thromboembolic events, and other clinically significant adverse events), and, if available, early ambulation status and discharge disposition.

The primary outcome reflects early in-hospital functional recovery and does not capture long-term post-discharge outcomes.

Complications were defined as clinically documented adverse events requiring medical intervention during hospitalization, including pneumonia, urinary tract infection, thromboembolic events, and other clinically significant complications, as defined by standardized institutional reporting protocols.

### 2.6 Statistical analysis

The statistical analysis was conducted using SPSS version 22 (IBM Corporation, Armonk, NY, USA). Continuous variables are reported as mean ± standard deviation or median (interquartile range), based on their distribution. Categorical variables are reported as frequencies and percentages.

The Shapiro–Wilk test was used to assess normal distribution. Group comparisons were performed by use of independent *t*-tests or Mann–Whitney U-tests for continuous variables and chi-square or Fisher's exact tests for categorical variables.

To investigate the independent relationships between frailty and sarcopenia and functional recovery (Barthel index), multivariable linear regression analysis was performed, controlling for age, sex, BMI, and comorbidity status.

Multicollinearity was assessed using variance inflation factors (VIF), and none were significantly correlated.

A statistically significant two-tailed *p*-value was determined to be ≤ 0.05.

### 2.7 Ethics statement

Approval for this research was granted by the Ethics Committee at the Department of Orthopedics at Shanxi Bethune Hospital, Shanxi Academy of Medical Sciences, and the Third Hospital of Shanxi Medical University (Approval No. YXLL-2025-201). As the study is retrospective and involved using de-identified patient records, written informed consent from patients was not required by the Institutional Ethics Committee. The research was conducted in accordance with the Declaration of Helsinki and other applicable guidelines.

## 3 Results

### 3.1 Patient selection

We initially assessed 150 patients with osteoporotic vertebral compression fractures for eligibility to participate in the study. Supplementary Figure S1 provides representative radiographic images of the osteoporotic vertebral compression fracture pattern. After applying the exclusion criteria as outlined, 30 patients were excluded due to high-energy trauma, pathologic fractures, neurological deficit, prior spinal surgery, and/or incomplete clinical data. Therefore, 120 patients were included in the analysis.

**Figure 1:**
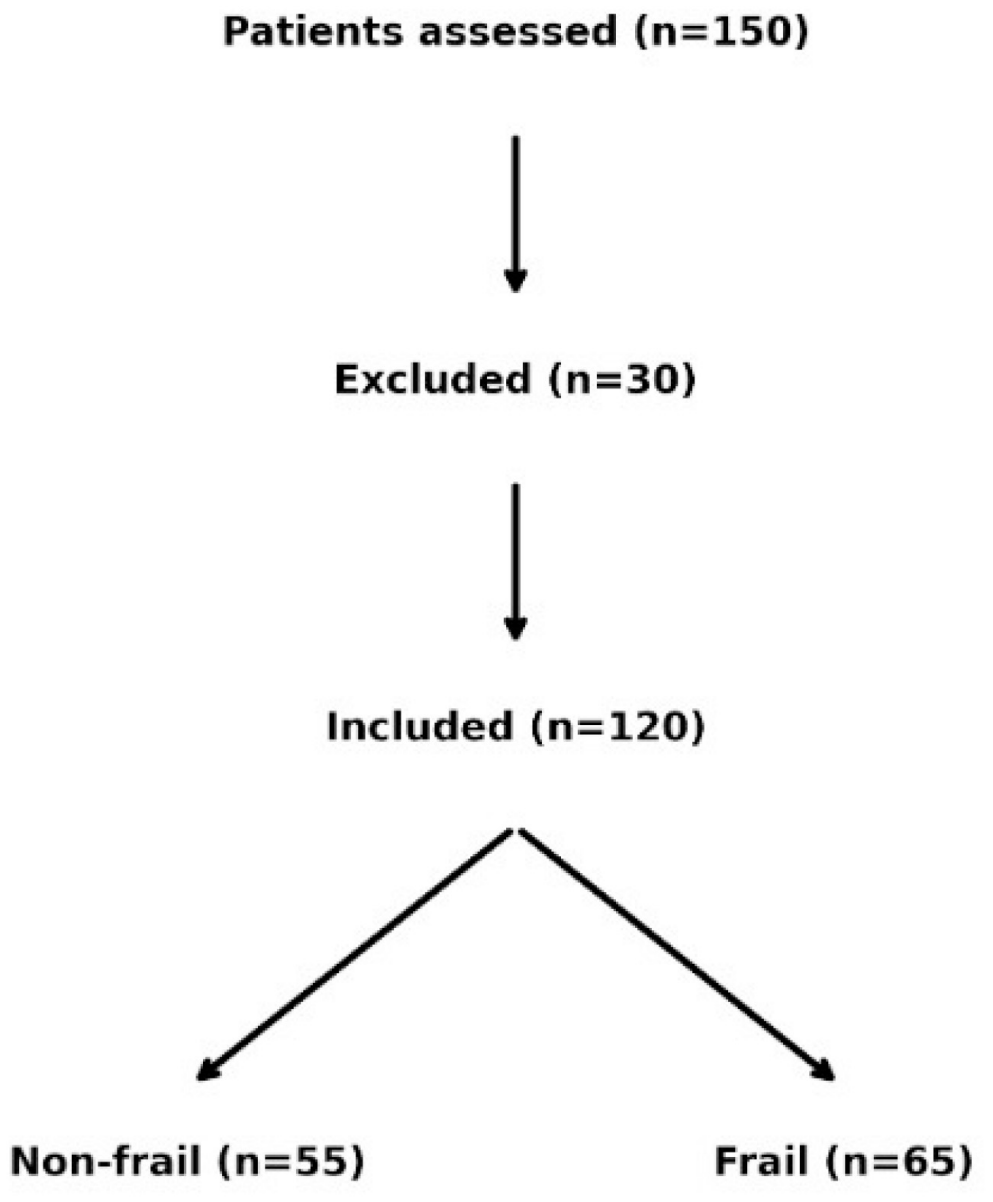
Flowchart of patient selection and study cohort formation. The screening procedure, exclusion criteria, and final inclusion protocol for patients diagnosed with an osteoporotic vertebral compression fracture are shown in this flowchart. The total number of patients included in this study was 120, and each was classified based on their frailty assessment. This figure summarizes the formation of the analytical cohort used in this research.

Of the 120 eligible patients, 55 were classified as non-frail and 65 as frail according to our frailty assessment criteria (Figure 1).

### 3.2 Baseline characteristics

Demographics and clinical characteristics of study participants (Table 1) were assessed for age, sex (female), height, weight, body mass index (BMI), and the presence of hypertension and diabetes. Cohorts were similar in average ages, with no statistically significant difference; however, a trend toward higher age in frail patients was observed (non-frail: 73.9 ± 5.8 years vs. frail: 75.6 ± 6.4 years; *p* = 0.08).

**Table 1:** Baseline characteristics of patients.

Variable	Total (*n* = 120)	Non-frail (*n* = 55)	Frail (*n* = 65)	*p*-value
Age, (years)	74.8 ± 6.2	73.9 ± 5.8	75.6 ± 6.4	0.08
Female, *n* (%)	62 (51.7%)	28 (50.9%)	34 (52.3%)	0.87
BMI, (kg/m^2^)	24.1 ± 3.0	24.5 ± 2.8	23.8 ± 3.2	0.21
Hypertension, *n* (%)	58 (48.3%)	25 (45.5%)	33 (50.8%)	0.56
Diabetes, *n* (%)	36 (30.0%)	14 (25.5%)	22 (33.8%)	0.32
Sarcopenia, *n* (%)	58 (48.3%)	20 (36.4%)	38 (58.5%)	0.01

Continuous variables are presented as mean ± standard deviation; categorical variables as *n* (%).

No statistically significant differences were found for BMI (24.5 ± 2.8 kg/m^2^ vs. 23.8 ± 3.2 kg/m^2^; *p* = 0.21), the prevalence of hypertension (45.5% vs. 50.8%; *p* = 0.56), or the prevalence of diabetes mellitus (25.5% vs. 33.8%; *p* = 0.32) between non-frail and frail subjects.

The distribution of treatment modalities between the frailty and sarcopenia groups did not differ significantly; however, its potential influence was considered in interpreting the findings.

However, the prevalence of sarcopenia in frail subjects (58.5%) was statistically significantly greater than in non-frail subjects (36.4%); *p* = 0.01.

### 3.3 Clinical outcomes according to frailty status

**Figure 2:**
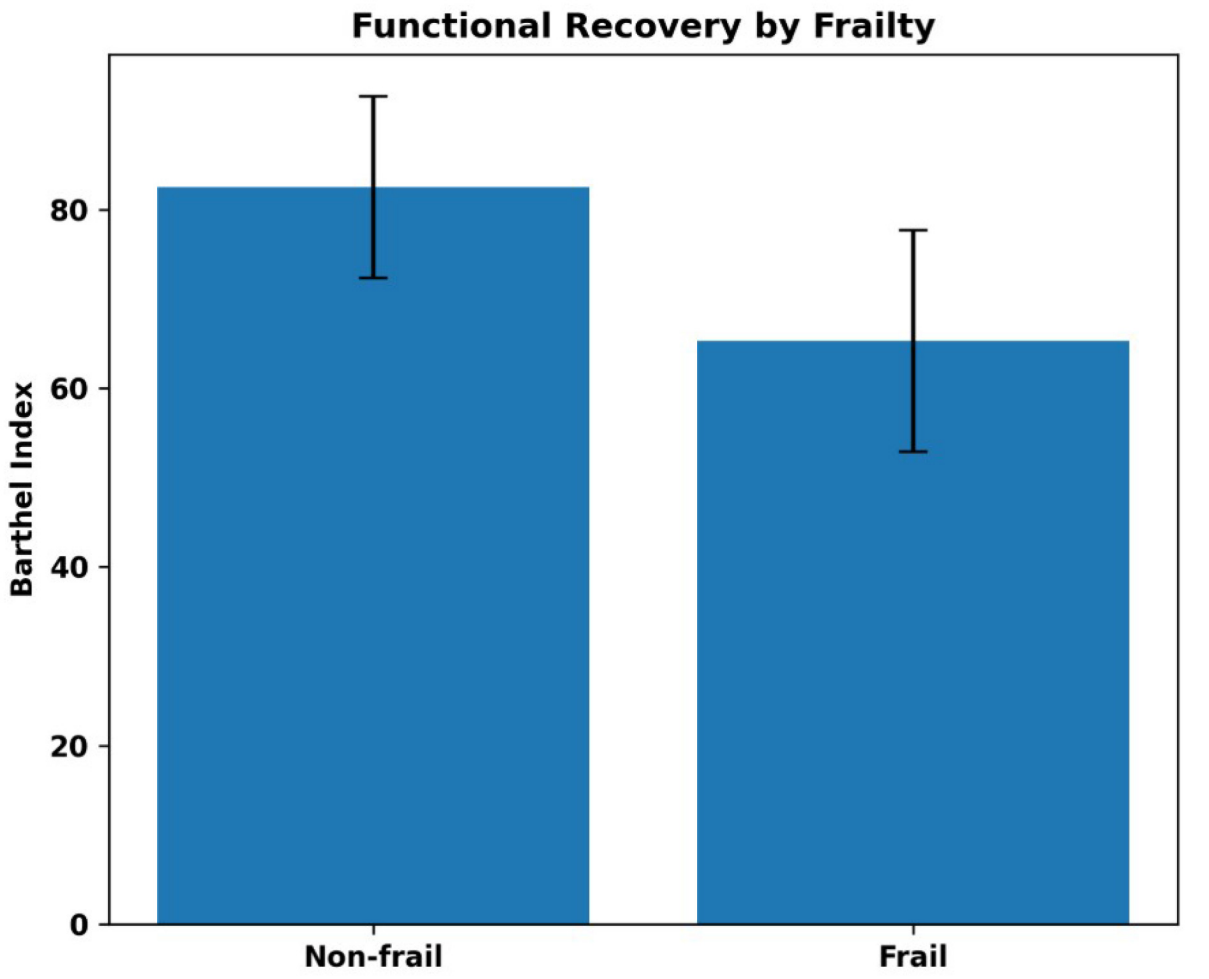
Comparison of functional recovery between frailty groups. Bar chart illustrating the difference in Barthel Index scores between frail and non-frail patients at discharge. Error bars represent standard deviation.

Clinical outcomes for frail and non-frail patients are shown in Table 2 and in Figure 2. Frail patients had significantly lower Barthel index scores at discharge (65.3 ± 12.4) than non-frail patients (82.5 ± 10.2) (*p* < 0.001). The intensity of pain, as measured using the Visual Analog Scale (VAS), was significantly higher among frail patients than non-frail patients (5.1 ± 1.5 vs. 3.8 ± 1.2) (*p* = 0.02).

**Table 2:** Clinical outcomes according to frailty status.

Outcome	Non-frail (*n* = 55)	Frail (*n* = 65)	*p*-value
Barthel index	82.5 ± 10.2	65.3 ± 12.4	< 0.001
VAS Score	3.8 ± 1.2	5.1 ± 1.5	0.02
Length of stay (days)	8.5 ± 3.2	12.4 ± 4.1	0.01
Complications, *n* (%)	6 (10.9%)	18 (27.7%)	0.03

Continuous variables are presented as mean ± standard deviation; categorical variables as *n* (%).

Frail patients had a significantly longer length of stay compared to their counterparts (12.4 ± 4.1 days vs. 8.5 ± 3.2 days) (*p* = 0.01), and they had a greater incidence of in-hospital complications compared to non-frail patients (27.7% vs. 10.9%) (*p* = 0.03).

### 3.4 Clinical outcomes according to sarcopenia status

The outcomes for patients with and without sarcopenia are summarized in Table 3. Sarcopenic patients exhibited significantly lower Barthel Index scores than non-sarcopenic patients (66.4 ± 11.7 vs. 80.2 ± 9.8) (*p* < 0.001), while patients with sarcopenia had greater visual analog scale (VAS) pain scores than non-sarcopenic patients (5.0 ± 1.4 vs. 3.9 ± 1.1) (*p* = 0.03). The mean duration of hospital stay for sarcopenic patients (12.0 ± 4.2 days) was greater than that of non-sarcopenic patients (9.1 ± 3.0 days) (*p* = 0.02). The sarcopenia group also had a greater incidence of complications than the non-sarcopenia group (27.6% vs. 12.9%) (*p* = 0.04).

**Table 3:** Clinical outcomes according to sarcopenia status.

Outcome	Non-sarcopenia (*n* = 62)	Sarcopenia (*n* = 58)	*p*-value
Barthel index	80.2 ± 9.8	66.4 ± 11.7	< 0.001
VAS score	3.9 ± 1.1	5.0 ± 1.4	0.03
Length of stay (days)	9.1 ± 3.0	12.0 ± 4.2	0.02
Complications (%)	8 (12.9%)	16 (27.6%)	0.04

Continuous variables are presented as mean ± standard deviation; categorical variables as *n* (%).

### 3.5 Multivariable analysis

**Figure 3:**
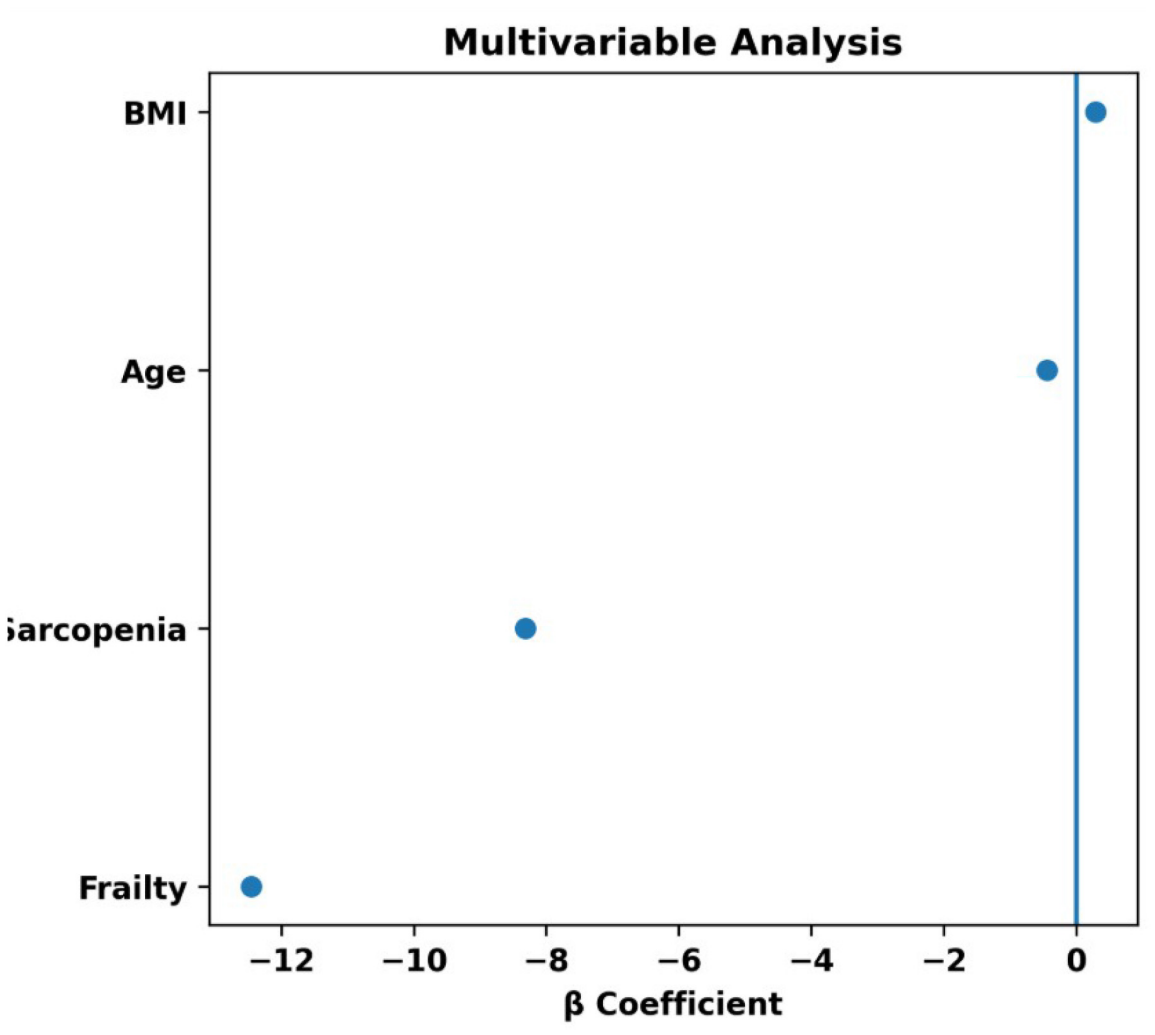
Multivariable regression analysis of factors associated with functional recovery. The forest plot illustrates the association of each variable with functional recovery, as measured by the Barthel Index. Both frailty and sarcopenia were negatively associated with functional recovery, while the effects of other covariates were comparatively smaller. The vertical reference line represents no effect.

The multivariable linear regression analysis outcomes for functional improvement (Barthel Index) are shown in Table 4 and depicted in Figure 3.

**Table 4:** Multivariable regression analysis for functional recovery.

Variable	β coefficient	Standard error	*p-*value
Frailty	−12.45	3.21	< 0.001
Sarcopenia	−8.32	2.85	0.004
Age	−0.45	0.18	0.01
BMI	0.28	0.15	0.07
Comorbidities	−3.12	1.45	0.03

Dependent variable: Barthel Index. β represents the unstandardized regression coefficient; standard error is reported for each estimate.

When evaluated in the multivariable model, frailty was associated with lower Barthel Index scores (β = −12.45; SE = 3.21; *p* < 0.001), while sarcopenia was significantly associated with lower functional recovery (Barthel Index scores) (β = −8.32; SE = 2.85; *p* = 0.004).

While there was a small but statistically significant inverse relation between age and functional improvement (β = −0.45; SE = 0.18; *p* = 0.01), comorbidity had an independent effect on the Barthel index (β = −3.12; SE = 1.45; *p* = 0.03).

Body mass index was not significantly associated with functional recovery (β = 0.28; SE = 0.15; *p* = 0.07).

## 4 Discussion

### 4.1 Main findings

The findings of this retrospective cohort study demonstrate that both frailty and sarcopenia were independently associated with reduced early functional recovery during hospitalization in older adults with osteoporotic vertebral compression fractures (OVCFs). Patients classified as frail exhibited significantly lower Barthel Index scores, higher pain levels, longer hospital stays, and increased complication rates compared to non-frail individuals. Similarly, patients with sarcopenia demonstrated adverse functional and clinical outcomes consistent with those observed in frail patients.

The associations remained statistically significant when adjusting for confounding factors, indicating that sarcopenia and frailty were independent predictors of early recovery. The results indicate that recovery from an OVCF is influenced not only by fracture characteristics but also by an individual's systemic physiological vulnerability and overall muscle health (1–6).

### 4.2 Comparison with previous studies

The findings from this study support the existing literature, which demonstrates that OVCFs represent skeletal damage and indicate physical decline and systemic frailty among older adults (1–8). Such fractures not only produce pain, reduced mobility, and loss of independence but also have a broad range of recovery patterns contingent on how individual patients respond (2–6).

This study also builds upon previous research by showing the association of frailty with sarcopenic conditions to functional outcomes during the initial hospitalization stay.

The relationship between poor outcomes following fragility fractures and frailty has been extensively researched. Several previous studies have established that frail patients experience a greater incidence of peri-hospital complications; therefore, they also require longer periods to recover to a functional level and are at risk for losing their independence as well (13–15). Additionally, there has been ample evidence indicating that there exists a bidirectional relationship between frailty and osteoporotic fractures in which fragility fractures accelerate the progression of frail patients, and frail patients are at an increased risk of sustaining osteoporosis-related fractures, and experience a higher incidence of complication rates after sustaining such injuries (14). This study supports the previously established framework.

Sarcopenia has demonstrated similar associations regarding the occurrence of impaired mobility, increased risk of fracture, and negative rehabilitation or recovery outcomes (16–18). Previous studies have demonstrated that following sustaining a vertebral fracture, patients with sarcopenia had higher complication rates and delayed recovery to a functional level compared with those patients who did not describe being sarcopenic (16–18). The current study extends these findings by demonstrating that sarcopenic patients experienced a statistically significantly lower-than-average functional recovery (as measured by Barthel Index scores) during the initial hospitalization period compared with their non-sarcopenic peers.

Although frailty and sarcopenia are often examined separately, they commonly co-occur; hence, their co-occurrence may signify a higher-risk phenotype (osteosarcopenic frailty) and may ultimately lead to worse outcomes due to their combined effects. Future studies examining the presence of combined phenotypes will enable researchers to develop improved risk stratification tools (19–23).

### 4.3 Mechanisms

The observed connections among frailty, sarcopenia, and delayed recovery is likely influenced by multiple factors and processes, including physiologic reserve and the ability of physiological, musculoskeletal, and metabolic functions to interact in complex ways.

Frailty is characterized by diminished physiologic reserve and an impaired response to stress due to multisystem failure (13–15, 24, 25). The frail patient is also more likely to develop post-operative complications such as deconditioning, infection, and delirium, all of which prolong the length of stay (LOS) in acute care and delay recovery.

Sarcopenia is associated with functional impairment directly. Lower levels of muscle mass and strength affect balance, trunk stability, and mobility, all of which are integral to recovery after vertebral fractures (16–20). Further, less functional muscle results in less successful rehabilitation interventions.

Within the setting of OVCFs, paravertebral muscle degeneration and reduced spinal extensor strength may contribute to kyphotic deformity progression, increased pain, and impaired mobility, thereby amplifying the impact of sarcopenia on functional recovery.

Sarcopenia is also linked to metabolic dysfunction. Notably, BMI did not show a significant association with functional recovery in this study, which may reflect its limited ability to distinguish muscle mass from adiposity, particularly in older adults with altered body composition. Nutritional deficits such as protein deficiency, anabolic resistance, and chronic low-grade inflammation will result in impaired muscle tissue regeneration and loss of physical function (19–23, 26, 27).

Although nutritional status is closely linked to sarcopenia, direct nutritional assessment (e.g., albumin levels) was limited in this study, and incomplete data availability precluded formal analysis. Future studies should further explore the role of nutritional status in functional recovery.

Frailty and sarcopenia have synergistic effects as both share common pathways, including inflammation and dysregulation of hormones, which then result in cumulative impairments in an individual's capacity to recover.

### 4.4 Clinical implications

There are clinical implications in these findings. Performing routine assessments of sarcopenia and frailty at admission may help identify patients at high risk of poor outcomes and ultimately enable early intervention through personalized care planning, including strategies for early mobilization, closer observation, and improved discharge planning.

Care planning for frail patients can be aided by regular assessments, which inform decisions about individualized care, as described above. Additionally, Sarcopenia is a modifiable condition that is a target for interventions with recovery and supportive care.

Patients with OVCFs will likely benefit from the use of multidisciplinary care teams that include orthopedic management, assessment by a geriatrician, and rehabilitation services (9–11, 28–38).

When considering how to best rehabilitate someone after an OVCF, rehabilitation should focus on strength training, functional goals specific to that person, and mobility.

In addition to being able to improve outcomes for these patients following admission to a hospital through a multidisciplinary approach, early identification of at-risk patients supports secondary prevention strategies such as fracture liaisons and pathways for coordinated post-fracture care, both of which have been shown to improve outcomes and reduce risk for future fractures (9–11). The increased complication rates observed in frail and sarcopenic patients were primarily related to medical complications such as infections and reduced mobility-related events, which may partly explain the prolonged hospitalization observed in these groups.

### 4.5 Limitations

There are several limitations in this study. Firstly, because it was designed as a retrospective study, it cannot establish causation and may be subject to selection bias. Secondly, the study's single-center design limits the generalizability of its results beyond the institution where it was conducted. Thirdly, the outcome measure of interest was discharge functional recovery (Barthel Index), which, although widely used, may not fully capture the complexity of functional dependence and rehabilitation needs in patients with OVCFs. Fourthly, treatment heterogeneity (including conservative vs. vertebral augmentation) was not accounted for in the multivariable model, which may affect overall clinical outcomes. Fifthly, fracture-specific variables (e.g., vertebral level, number of fractured segments, and degree of deformity) were excluded from the multivariable model due to inconsistent data availability. Sixthly, the limited availability of structured nutritional data, including albumin across all patients, may have reduced the ability to fully explore its relationship with sarcopenia and recovery.

Finally, unmeasured confounding factors, including variability in rehabilitation intensity and other clinical parameters, may have influenced the study findings. In addition, sensitivity analyses excluding patients classified solely on surrogate indicators were not performed due to sample size constraints; this should be considered when interpreting the results.

## 5 Conclusion

This cohort study of older adults with osteoporotic vertebral compression fractures (OVCFs) demonstrates that frailty and sarcopenia were independently associated with reduced early functional recovery during hospitalization. Patients with these conditions experienced lower functional independence, higher pain levels, longer hospital stays, and increased complication rates compared to those without frailty or sarcopenia.

These findings highlight the importance of incorporating geriatric and muscle health assessments into routine clinical evaluation to improve risk stratification and guide individualized care.

However, given that the primary outcome reflects short-term in-hospital recovery and considering the retrospective single-center design and potential confounding factors, these findings should be interpreted with caution.

Further prospective, multicenter studies with long-term follow-up are required to confirm these associations and to evaluate whether targeted interventions addressing frailty and sarcopenia can improve sustained functional outcomes in this population.

## Data Availability

The original contributions presented in the study are included in the article/Supplementary material, further inquiries can be directed to the corresponding author.

## Supplementary material

SUPPLEMENTARY FIGURE S1 Representative radiographic image of osteoporotic vertebral compression fracture. Representative lateral **(A)** and anteroposterior **(B)** radiographs demonstrating osteoporotic vertebral compression fracture in an elderly patient. The images illustrate typical vertebral body height loss and structural deformity consistent with OVCFs. This figure provides a visual reference for the type of fractures included in the study.
