# Supplementary material for: Frailty and sarcopenia as independent predictors of early functional recovery in older adults with osteoporotic vertebral compression fractures: a retrospective cohort study
Source: Front Nutr. 2026 Jun 9;13:1841245. doi: 10.3389/fnut.2026.1841245 (PMC13286839; doi:10.3389/fnut.2026.1841245)
Supplement: Supplementary file 1 [file Table_1.docx]

**STROBE Statement—checklist of items that should be included in reports of observational studies**

| **Item** | **Page/Section** | **Description** |
| --- | --- | --- |
| 1(a) | Title | Retrospective cohort stated |
| 1(b) | Abstract | Structured summary provided |
| 2 | Introduction | Background described |
| 3 | Introduction | Objectives stated |
| 4 | Methods 2.1 | Study design stated |
| 5 | Methods 2.1 | Setting and dates |
| 6 | Methods 2.2 | Participants criteria |
| 7 | Methods 2.3–2.5 | Variables defined |
| 8 | Methods 2.3 | Data sources EMR |
| 9 | Methods 2.6 | Bias addressed |
| 10 | Methods 2.2 | Sample size |
| 11 | Methods 2.6 | Variables handled |
| 12 | Methods 2.6 | Statistical analysis |
| 13 | Results 3.1 | Participants flow |
| 14 | Results 3.2 | Baseline data |
| 15 | Results 3.3–3.5 | Outcome data |
| 16 | Results 3.5 | Main results |
| 17 | Results | Additional analyses |
| 18 | Discussion 4.1 | Key results |
| 19 | Discussion 4.5 | Limitations |
| 20 | Discussion | Interpretation |
| 21 | Discussion | Generalisability |
| 22 | Funding | No funding |
