# Supplementary figures and images for: Frailty and sarcopenia as independent predictors of early functional recovery in older adults with osteoporotic vertebral compression fractures: a retrospective cohort study
Source: Front Nutr. 2026 Jun 9;13:1841245. doi: 10.3389/fnut.2026.1841245 (PMC13286839; doi:10.3389/fnut.2026.1841245)

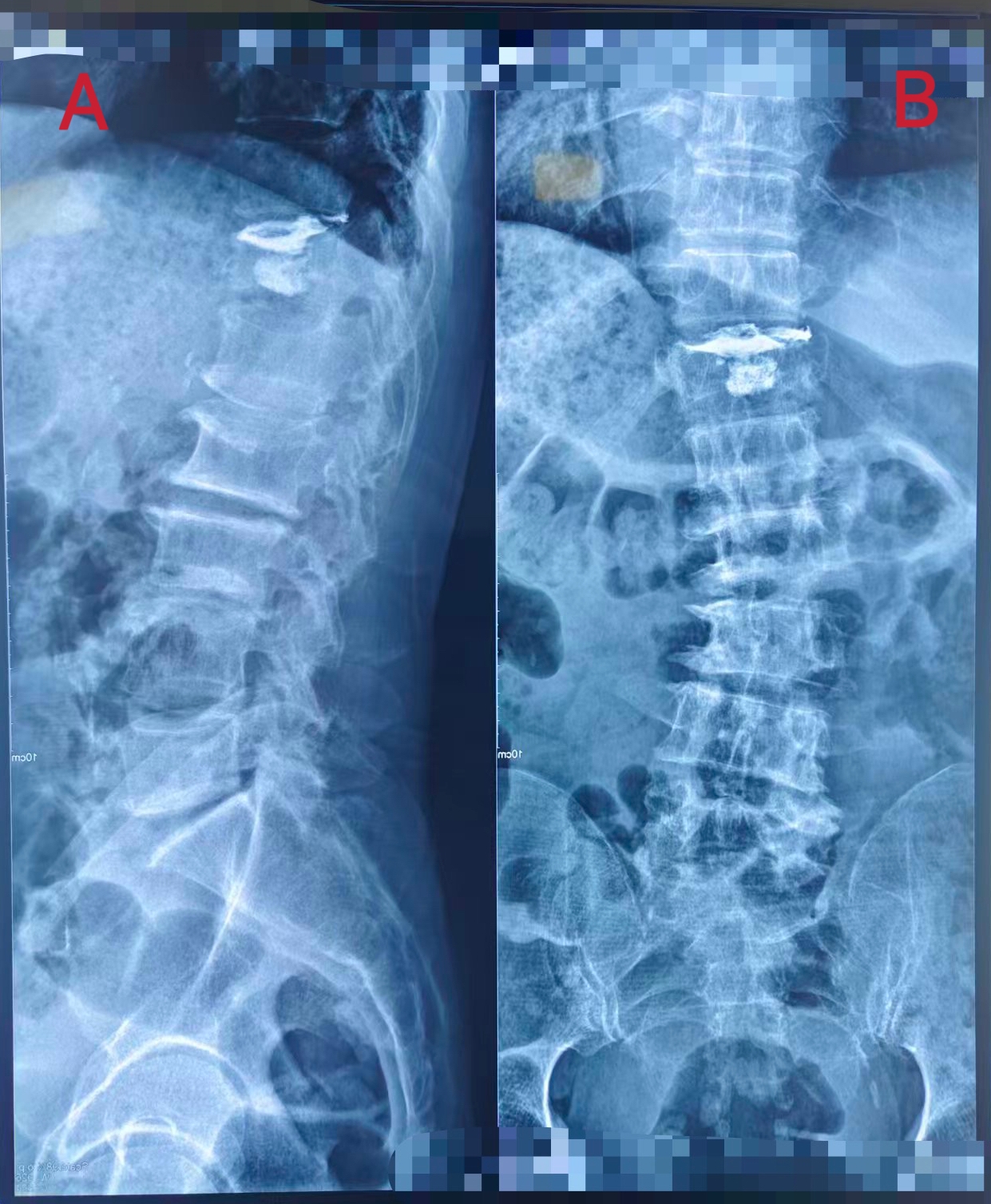

Supplement: SUPPLEMENTARY FIGURE S1 — Representative radiographic image of osteoporotic vertebral compression fracture. Representative lateral (A) and anteroposterior (B) radiographs demonstrating osteoporotic vertebral compression fracture in an elderly patient. The images illustrate typical vertebral body height loss and structural deformity consistent with OVCFs. This figure provides a visual reference for the type of fractures included in the study. [file Image_1.jpg]
